# TOP2A Expression in Pheochromocytoma and Abdominal Paraganglioma: a Marker of Poor Clinical Outcome?

**DOI:** 10.1007/s12022-022-09746-w

**Published:** 2023-01-19

**Authors:** Karolina Solhusløkk Höse, Adam Stenman, Fredrika Svahn, Catharina Larsson, C. Christofer Juhlin

**Affiliations:** 1 Department of Oncology-Pathology, Karolinska Institutet, Stockholm, Sweden; 2 Department of Molecular Medicine and Surgery, Karolinska Institutet, J6:20 BioClinicum Karolinska University Hospital, 171 64 Solna, Sweden; 3 Department of Breast, Endocrine Tumours and Sarcoma, Karolinska University Hospital, Solna, Sweden; 4 Department of Clinical Pathology and Cancer Diagnostics, Karolinska University Hospital, Solna, Sweden

**Keywords:** Pheochromocytoma, Paraganglioma, TOP2A, Prognostic marker, Metastasis

## Abstract

Pheochromocytoma and abdominal paraganglioma (PPGL) are rare neuroendocrine tumors originating from chromaffin cells. Even though only 10–15% of the tumors metastasize, all PPGLs are considered potentially malignant. Topoisomerase 2A (TOP2A) is a protein involved in cell proliferation and has been found to be over-expressed in metastatic PPGL. To provide support whether TOP2A could serve as a prognostic marker, 88 PPGLs (of which 8 metastatic/relapsing) and 10 normal adrenal gland samples were assessed for *TOP2A* mRNA expression using quantitative real-time PCR (qRT-PCR) and TOP2A immunohistochemistry. Comparisons to clinical parameters connected to metastatic behavior were made, and The Cancer Genome Atlas was used for validation of the results. A significant association between high *TOP2A* mRNA expression in primary PPGL and subsequent metastatic events (*p* = 0.008) was found, as well as to specific histological features and clinical parameters connected to metastatic behavior and mutations in *SDHB*. TOP2A immunoreactivity was calculated as an index of positive nuclei divided by the total amount of nuclei, and this index associated with *TOP2A* mRNA levels (*p* = 0.023) as well as the Ki-67 labeling index (*p* = 0.001). To conclude, TOP2A is a potential prognostic marker as it is frequently elevated in PPGL displaying subsequent metastatic disease, and future studies in larger cohorts are warranted to determine if a TOP2A index as assessed by immunohistochemistry could be a marker of poor outcome. Additionally, elevated levels of TOP2A could indicate a potential actionable event, and future studies with topoisomerase inhibitors would be of interest.

**Supplementary Information:**

The online version contains supplementary material available at 10.1007/s12022-022-09746-w.

## Introduction

Pheochromocytoma (PCC) and abdominal paraganglioma (aPGL), mutually referred to as “PPGL”, are rare, neuroendocrine tumors affecting 6.6 persons per million [1]. Metastases to non-chromaffin sites, such as bone and regional lymph nodes, occur in 10–15% of PCC and 30–50% of aPGL [2, 3]. The first-hand treatment for all PPGL is surgery, which is curative for most patients [4], although patients with spread disease often exhibit limited responses to chemotherapy and thereby have a poor prognosis [5]. Additionally, there is no fail-proof way to predict future metastases, neither pre- nor post-operatively, and therefore it is stated that all PPGLs are considered potentially malignant [2]. This has resulted in recommendations for life-long follow-up of all patients [6], regardless of the relatively small risk of metastatic disease. An indication of the malignant potential can be obtained from the genetic background, and histological and immunohistochemical characteristics, as well as certain clinical parameters [7]. PPGL is one of the most hereditable tumor types, with 40% of the patients exhibiting a constitutional mutation in a PPGL susceptibility gene [8, 9], where mutations in a pseudo-hypoxia-related gene such as *succinate dehydrogenase complex, subunit B* (*SDHB*) indicate a higher risk of metastases (“Cluster 1” PPGL) compared to PPGL with kinase-associated mutations (“Cluster 2” PPGL) [2]. Additionally, several histological grading systems such as The Pheochromocytoma of the Adrenal gland Scaled Score (PASS) and The Grading System for Adrenal Pheochromocytoma and Paraganglioma (GAPP) have been put forward in attempts to distinguish metastatic tumors [2]. Even though both PASS and GAPP may have sub-optimal positive predictive values and are currently not endorsed by the 2022 WHO classification, the GAPP score in particular has been reproduced in independent studies [3, 10, 11]. Furthermore, several clinical parameters such as low age at diagnosis, male sex, norepinephrine production, larger tumor size, Cluster 1 (pseudo-hypoxic) phenotype, and extra-adrenal origin (aPGL) have been associated with an increased risk of metastases in other studies [11–13].

Given the general absence of clinical, histological, and molecular markers that can identify and separate all potentially metastatic PPGLs from non-metastatic ones, researchers are focusing their efforts into a clinically useful predictor of disseminated disease. In a recent study from our group, several differentially expressed genes were identified when comparing mRNA transcriptional profiles between metastatic and non-metastatic PPGL [14]. Of note, Topoisomerase type II alpha (*TOP2A*) was found over-expressed in the metastatic cases and could therefore in theory constitute a possible prognostic marker to identify patients with a potentially metastatic tumor. TOP2A is part of the topoisomerase protein family that facilitate replication and the ability of malignant tumors cells to undergo infinite proliferation [15], and topoisomerase inhibitors are used in the clinic for treating several other types of cancer by inhibiting this mechanism [16]. Topoisomerases reversibly relax/unwind DNA strands enabling DNA replication, recombination, and transcription, thereby contributing to cell proliferation [17]. Topoisomerase type II is active in several metabolic mechanisms, such as chromosome segregation in mitosis. It consists of TOP2A and topoisomerase type II beta (TOP2B), in which TOP2A regulates cell proliferation and TOP2B regulates gene transcription. TOP2A is over-expressed in several types of tumors, including carcinomas of adrenocortical, nasopharyngeal, and gallbladder origin, as well as breast, ovary, and lung cancer [18, 19]. Additionally, over-expression of TOP2A has been associated with aggressive tumor types, tumor recurrence, and poorer survival [20, 21], thereby indicating the potential of TOP2A as a prognostic marker.

Of note, previous findings of *TOP2A* mRNA over-expression in PPGL have been reported [22–25], in some of which associations with poorer prognosis were observed. Also, a recent effort using single cell sequencing data found that *TOP2A* and *MKI67* mRNA levels were elevated in metastatic PPGLs with aberrancies in the tricarboxylic acid (TCA) cycle [26]. In terms of immunohistochemical studies, TOP2A proliferation index has been found to correlate to Ki-67 immunoreactivity, and TOP2A was also elevated in PPGLs denoted as malignant according to earlier classification systems [27]. Given these former observations, a reappraisal of TOP2A expression in a well-defined cohort of PPGL with updated definitions of metastasis as per the 2022 WHO classification could be of potential clinical utility considering the strong association with worse outcome in addition to the ever-changing landscape of personalized cancer medicine and development of topoisomerase inhibitors in clinical practice. We therefore sought to investigate TOP2A expression and correlate it to genotype, histological risk factors as defined by the PASS score, the Ki-67 labeling index, and clinical outcome.

## Materials and Methods

### Study Cohort

The study cohort consisted of PPGL primary tumor samples (classified according to the 2017 WHO Classification of Endocrine Tumors [28], with the definition of metastasis collected from the novel 2022 WHO Classification) [3], and non-tumoral adrenal tissue samples surgically removed at the Karolinska University Hospital, Stockholm, Sweden. Tissues were snap frozen in liquid nitrogen and kept in − 80 °C until further analysis. The tumors were stored and collected at the endocrine biobank at the Karolinska University Hospital. The inclusion criteria consisted of histologically verified PPGL with adequate tissue content to allow molecular studies. Cases that did not fulfil these criteria were excluded from the study. For extraction of RNA, fresh frozen tissue was used. Representativity testing showed that the tissue samples were homogenous including mostly tumor cells, in addition to sustentacular cells and endothelial cells. The proportion of tumor cells in the tissue samples had a median value between 70 and 80% (data not shown). Paraffin-embedded tumor tissue samples were used for immunohistochemistry. All tissue samples were collected with informed consent and the study of the tissue material was approved by the Swedish Ethical Review Authority.

The final study cohort included 88 PPGLs (78 PCC and 10 aPGL, Supplementary Table 1) and 10 normal, de-identified adrenal samples taken adjacent to tumors. The PPGL cohort included 47 females and 41 males, with a median age at diagnosis of 53.4 years with a range from 14 to 83 years (Supplementary Table 1). The tumors have been previously screened for mutations in the PPGL disease genes *EGLN1*, *EPAS1*, *KIF1Bβ*, *MAX*, *MEN1*, *NF1*, *RET*, *SDHA*, *SDHB*, *SDHC*, *SDHD*, *SDHAF2*, *TMEM127*, and *VHL* [14]. The clinical parameters sex, age, tumor size, metastasis or relapse, follow-up time, absolute and disease-related outcome, PASS score and individual parameters, biochemistry and Cluster 1 or 2 genotype were also known for these study samples [8, 14]. The division into Cluster 1 or 2 was based on previous studies and available data for mRNA expression profiles and mutations [29].

We determined malignant behavior in PPGL based on distant metastases or relapsing, grossly infiltrative disease. In this study, 7 cases had been diagnosed with metastases to non-chromaffin tissues, whereas a single, additional case exhibited recurrent disease with extensive infiltration of surrounding tissues. Six out of 7 metastatic cases were verified with histology/cytology, and one single case with disseminated bone metastases was verified using a highly specific 11C-meta-hydroxyephedrine PET scan. Moreover, 6 out of 7 metastatic PPGLs displayed lymph node and/or bone metastases, and only a single case displayed solitary liver metastases at diagnosis (Supplementary Table 1). However, as the latter case harbored a somatic *KMT2D/MLL2* mutation and the patient was negative for any credible susceptibility gene variant in germline, the occurrence of histologically verified pheochromocytoma cells in the liver strongly argued against the development of a primary paraganglioma at this location — and hence this case was believed to be a *bona fide* metastasis. Four patients in the cohort died due to their disease (Supplementary Table 1).

### Real-time Quantitative PCR (qRT-PCR) for Quantification of TOP2A mRNA Expression

Complementary DNA (cDNA) was converted from RNA, using High-Capacity cDNA Reverse Transcription Kit (Thermo Fisher, MA, USA). The protocol recommended by the manufacturer was followed and TaqMan 2 × universal PCR master mix was used together with 20 ng of cDNA for each sample. Assay ID Hs03063307_m1 was used for *TOP2A* and Hs00187842_m1 was used for *B2M* which served as an endogenous control. Quant Studio 7 (Thermo Fisher, MA, USA) was used for the analyses with each sample in three technical replicates. A mean value was calculated, and the relative expression was determined after normalization to *B2M* and calculations according to the 2^−ΔCt^ method. *TOP2A* mRNA expression was quantified for 83 PPGL (Supplementary Table 1) and 10 normal adrenal samples.

### The Cancer Genome Atlas (TCGA) Database

Data from the TCGA database was downloaded from https://www.cbioportal.org in October 2021. The PanCancer Atlas set was used and primary tumors of PCC and aPGL were selected [30]. Head and neck tumors were excluded, as well as tumors with missing values of clinical data. In total, 159 cases were selected (134 PCC and 25 aPGL), including 72 males and 87 females with a median age of 47 years. *TOP2A* mRNA levels were compared to clinical parameters such as sex, age at diagnosis, progression of disease, absolute outcome, and disease-related outcome. Additionally, data of potential mutations and copy numbers of the *TOP2A* gene were collected. Methylation density values for *TOP2A* were collected from UCSC Xena Functions Genomics Explorer (https://xenabrowser.net/) in October 2021.

### TOP2A Immunohistochemistry

Formalin-fixed paraffin-embedded blocks from 37 PPGL with representative tumor tissue were cut at 4 µm and deparaffinized in xylene, followed by rehydration in ethanol. Antigen retrieval was performed using a Decloaking Chamber (Biocare Medical, CA, USA) set for 5 min at 110 °C in citrate buffer pH 6 (Sigma-Aldrich, MO, USA, catalogue no. C-9999). Peroxidase blocking was performed by incubation in hydrogen peroxide, followed by BSA blocking (Sigma-Aldrich, MO, USA, catalogue no. A-4503). The TOP2A polyclonal antibody (PA5-110,707) was diluted 1:800 in Redior Red diluent (Biocare Medical, CA, USA, catalogue no. #PD9004M) overnight. For detection the Biocare Medical Mach-1 Universal HRP-Polymer Kit was used (catalogue no. MIU539L10) according to protocol. Furthermore, counterstaining with hematoxylin as well as rehydration in ethanol and xylene was performed. Control tissues included de-identified cases of a normal adrenal gland as a negative control and normal testicular tissue as a positive control. A total amount of 37 PPGL (of which 33 were informative for *TOP2A* mRNA status) were immunostained for TOP2A. Since TOP2A is a predominantly nuclear antigen associated with tumor cell proliferative activity, we incorporated an approach similar to the established Ki-67 labeling index, counting the number of positive nuclei in hotspot areas and dividing by the total number of tumor nuclei, counting at least 2000 cells using an ocular grid. This resulted in a TOP2A index for each case.

### Ki-67 Labeling Index

We scrutinized the pathology reports of all PPGL cases included in this study in which TOP2A immunohistochemical data was available and retrieved the clinical routine Ki-67 labeling index whenever available (*n* = 22; Supplementary Table 1). The protocol of the Ki-67 immunohistochemistry at our institution has been previously described in detail [31]. In short, for cases diagnosed between 1999 and 2002, the staining was manually performed by a small group of experienced lab technicians, whereas automated staining procedures were used from 2003 and onwards. From 1999 to 2015, the Mib-1 antibody clone (Immunotech, Marseille, France) was used as a primary antibody, but was replaced in 2016 by the CONFIRM anti-Ki-67 antibody (clone 30–9, Roche, Basel, Switzerland). The scoring methodology has been consistent by counting 2000 cells using hot spot areas and an ocular grid.

### Statistics

SPSS 27 (IBM SPSS, Armonk, NY, USA) was used for statistical analyses. Variables were divided into two groups: categorical (tissue type, sex, individual PASS parameters, metastasis or relapse, absolute outcome, disease-related outcome, Cluster 1 or 2 genotype, noradrenaline and adrenaline production) and numerical (age, tumor size, follow-up time, PASS score and *TOP2A* mRNA levels). Mann–Whitney *U*-test was used to compare groups. Spearman’s rank-order correlation was used for correlation analysis. Log rank test was used to compare survival between two groups and illustrated with Kaplan–Meier plots. PPGL patients were divided according to *TOP2A* mRNA levels (above or below mean). The data was visualized using a cumulative scale, which means that every event was treated independently. Fisher’s exact test was used to compare associations between two categorical variables. Multivariate analysis with linear regression was used to test different clinical parameters as independent variables in the perspective of predicting metastasis. *p-*values ≤ 0.05 were considered statistically significant.

## Results

### Comparisons Between TOP2A mRNA Expression and Clinical/Genetic Parameters

**Fig. 1:**
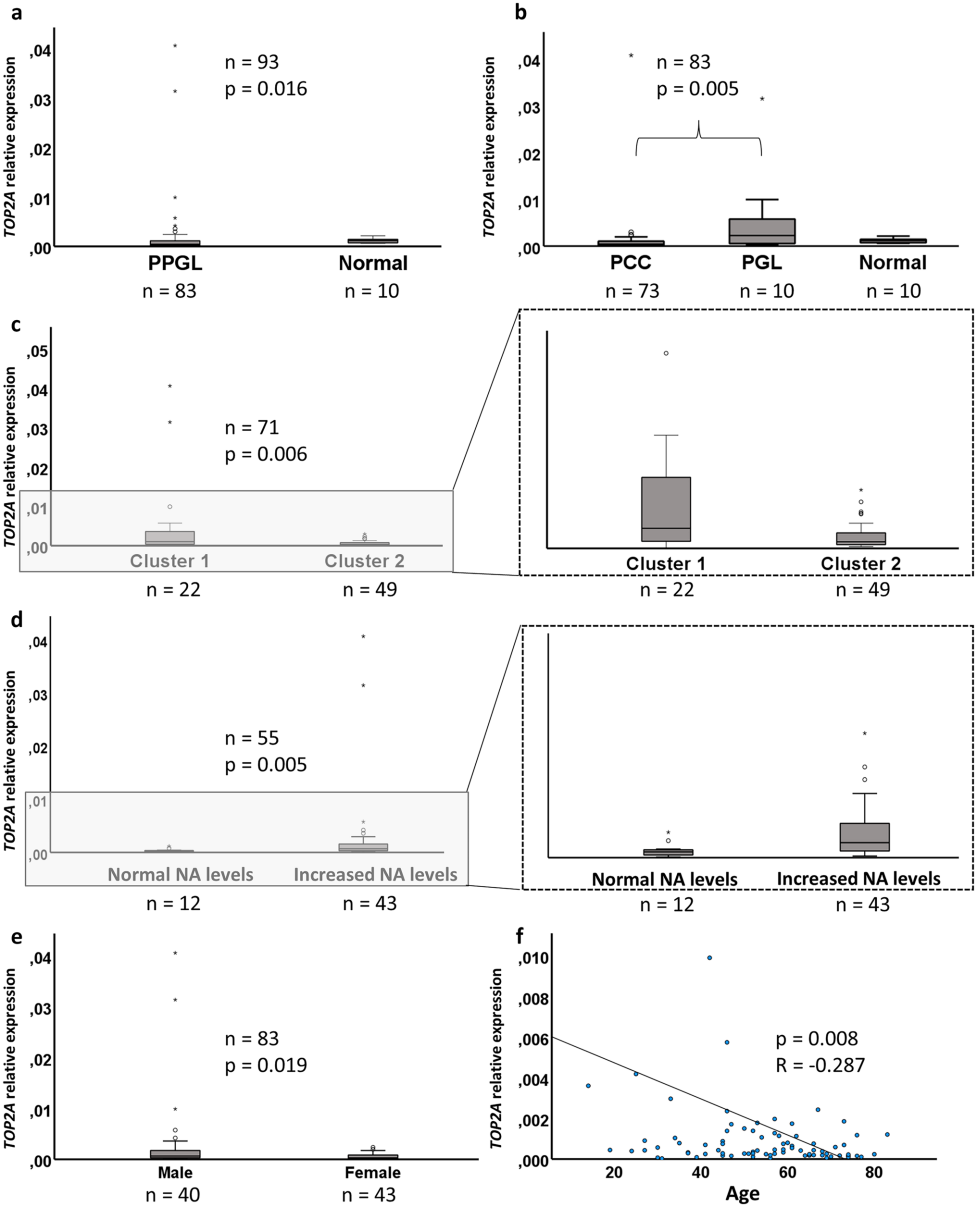
Associations between increased *TOP2A* mRNA expression and clinical parameters associated with malignant behavior in pheochromocytoma and paraganglioma (PPGL). **a** Relative expression of *TOP2A* in PPGL compared to expression in normal adrenal tissue. **b** Relative expression of *TOP2A* in the tissue type PCC compared to aPGL. *TOP2A* mRNA expression in normal adrenal tissue is also visualized. **c** Relative expression of *TOP2A* comparing Cluster 1 and 2. Enlargement without outliers to the right. **d** Relative expression of *TOP2A* in tumors from patients with normal levels of noradrenaline (NA) compared to increased levels. Enlargement without outliers to the right. **e** Relative expression of *TOP2A* in male sex compared to female sex. **f** Inverse relationship between *TOP2A* mRNA levels and age at diagnosis

Quantification of *TOP2A* mRNA in a cohort of primary PPGL and normal adrenal samples revealed a generally increased expression in PPGL as compared to normal adrenal (Fig. 1a). Additionally, higher relative expression of *TOP2A* mRNA was found in aPGL compared to PCC, while the lowest levels of *TOP2A* mRNA were observed in normal adrenal tissues (Fig. 1b).

Table 1 and Fig. 1 present data and statistical significance of several clinical parameters compared to expression of *TOP2A*, investigating PPGL as a group as well as aPGL and PCC separately. For PPGL, high expression of *TOP2A* mRNA was associated with the aPGL tumor type (as opposed to PCC), male sex (as opposed to female sex), metastasis or local recurrence (as opposed to no metastasis or local recurrence), Cluster 1 genotype (as opposed to Cluster 2 tumors), and high levels of noradrenaline (as opposed to normal levels). In addition, *TOP2A* mRNA was inversely correlated to patient age at diagnosis. These parameters have been associated with metastatic behavior in other studies [12, 32]. When analyzing aPGL and PCC separately, an association was only found between high expression of *TOP2A* mRNA and high noradrenaline levels in PCC (Table 1).

**Table 1:** Clinical and genetic parameters and statistical comparisons to *TOP2A* mRNA levels in PPGL, aPGL, and PCC separately

**Parameter**	***TOP2A*** **in PPGL**	***TOP2A*** **in aPGL**	***TOP2A*** **in PCC**
	***p*-value, observations**	***p*-value, observations**	***p*-value, observations**
*Type of tumor*	***p* = 0.005**		
PCC/aPGL	*n* = 73/*n* = 10		
*Sex*	***p* = 0.019**	*p* = 0.192	*p* = 0.148
Male/female	*n* = 40/*n* = 43	*n* = 8/*n* = 2	*n* = 32/*n* = 41
*Age at diagnosis*	***p* = 0.008**, *R* = − 0.287	*p* = 0.467, *R* = − 0.261	*p* = 0.114, *R* = − 0.187
Median (min–max) years	54.3 (14–83)	34.5 (14–73)	56 (19–83)
*Tumor size*	*p* = 0.644; *R* = 0.051	*p* = 0.144; *R* = − 0.497	*p* = 0.457; *R* = − 0.088
Median (min–max) mm	45 (15–160)	45 (30–70)	45 (15–160)
*Total PASS score*	*p* = 0.126; *R* = 0.193	*p* = 0.498; *R* = − 0.261	*p* = 0.426; *R* = 0.109
median (min–max)	3 (0–10)	5 (1–9)	2 (0–10)
*Noradrenaline (NA) levels*	***p* = 0.005**	*p* = 0.275	***p* = 0.012**
Increased NA/normal NA	*n* = 43/*n* = 12	*n* = 7/*n* = 1	*n* = 36/*n* = 11
*Adrenalin (A) levels*	*p* = 0.175	*p* = 0.456	*p* = 0.051
Increased A/normal A	*n* = 24/*n* = 32	*n* = 3/n = 5	*n* = 21/*n* = 27
*Cluster 1 or 2 genotype*	***p* = 0.006**	*p* = 0.079	*p* = 0.197
Cluster 1/Cluster 2	*n* = 22/*n* = 49	*n* = 7/*n* = 2	*n* = 15/*n* = 47
*SDHB genotype*	***p* < 0.001**	*p* = 0.183	
Mutation/wildtype	*n* = 3/*n* = 80	*n* = 3/*n* = 7	*n* = 0/*n* = 73
*TERT promoter genotype*	***p* = 0.048**	*p* = 0.2	
Mutation/wildtype	*n* = 1/*n* = 82	*n* = 1/*n* = 9	*n* = 0/*n* = 73
*Metastasis or local recurrence*	***p* = 0.008**	*p* = 0.055	*p* = 0.374
Metastasis	*n* = 7	*n* = 4	*n* = 3
No metastasis	*n* = 76	*n* = 6	*n* = 70
Absolute outcome*	*p* = 0.915	*p* = 0.201	*p* = 0.357
Dead	*n* = 20	*n* = 4	*n* = 16
Alive	*n* = 63	*n* = 6	*n* = 57
Absolute outcome**	*p* = 0.184		
Disease-related outcome*	*p* = 0.096	*p* = 0.296	*p* = 0.457
DOD	*n* = 4	*n* = 2	*n* = 2
Alive or DOC	*n* = 79	*n* = 8	*n* = 71
Disease-related outcome**	***p* = 0.050**		

Significant *p*-values (< 0.05) are highlighted in bold

*Mann Whitney U test

**Log Rank test

Levels of *TOP2A* mRNA were associated with *SDHB* mutation (*p* < 0.001, Mann–Whitney *U*) when comparing 3 cases with *SDHB* mutation and 80 *SDHB* wild-type cases, and with *TERT* promoter mutation (*p* = 0.048, Mann–Whitney *U*) when comparing one case with this mutation and 82 *TERT* promoter wild-type cases (Table 1). No other germline or somatic mutations were associated with *TOP2A* mRNA levels (Supplementary Table 1).

Data collected from the TCGA database was used to compare levels of *TOP2A* mRNA to clinical parameters in another cohort, to validate the results obtained in this study. The mRNA levels ranged from 14.3 to 1916.8 with a median value of 121.4 and a mean value of 187. *TOP2A* mRNA correlated inversely with the age at diagnosis; however, no significant associations were found when comparing tissue types or patient sex (Table 2).

**Table 2:** Clinical and genetic parameters and statistical comparisons to *TOP2A* mRNA levels in 159 PPGL from the TCGA dataset

**Parameter**	***p*-value, observations**
*Type of tumor*	*p* = 0.573
PCC/aPGL	*n* = 134/*n* = 25
*Sex*	*p* = 0.621
Male/female	*n* = 72/*n* = 87
*Age at diagnosis*	***p* < 0.001**, *R* = − 0.356
Median (min–max) years	46 (19–83)
Progression of disease*	***p* = 0.043**
Progression	*n* = 18
No progression	*n* = 141
Progression of disease**	*p* = 0.386
*Absolute outcome*	*p* = 0.082
Alive	*n* = 154
Dead	*n* = 5
*Disease-related outcome*	*p* = 0.078
Alive or DOC	*n* = 156
DOD	*n* = 3
*TOP2A* mutation	
Mutation	*n* = 0
Wild-type	*n* = 159
*TOP2A c*opy numbers	
Gain	*n* = 5
No alteration	*n* = 119
Loss	*n* = 33

Significant *p*-values (< 0.05) are highlighted in bold. Progression of disease = recurrence or progress of the tumor (defined by TCGA)

*Mann Whitney U test

**Log Rank test

Furthermore, associations between the individual histological PASS parameters and *TOP2A* mRNA expression in this study were examined (Table 3). For each PASS variable, the term “positive” indicates that the specific tumor scored positive for that variable, in contrast to the term “negative” indicating that the specific characteristic was not visualized in the histological examination. Cases were divided into two groups to distinguish between high and low expression of *TOP2A* with expression above or below the mean. The analyses revealed that high *TOP2A* expression was associated with the presence of tumor cell spindling (Fisher’s exact test, *p* = 0.013), mitotic figures (*p* = 0.042), and capsular invasion (*p* = 0.001) (Table 3). Furthermore, higher total PASS score was associated to higher risk of metastasis (*p* = 0.003, Mann–Whitney *U*), which has also been seen in previous studies based on this cohort [14].

**Table 3:** Comparison between PASS parameters and high or low *TOP2A* mRNA expression levels in PPGL

**PASS variable (informative cases,** ***n*** **= 70)**	**Low** ***TOP2A*** **(*n*** **=)**	**High** ***TOP2A*** **(*n*** **=)**	***p*-value**
*Large nests or diffuse growth*			*p* = 0.709
Positive	20	2	
Negative	41	7	
*Focal or confluent necrosis*			*p* = 0.055
Positive	9	4	
Negative	52	5	
*High cellularity*			*p* = 1.000
Positive	11	1	
Negative	50	8	
*Cellular monotony*			*p* = 0.219
Positive	5	2	
Negative	56	7	
*Tumor cell spindling*			***p* = 0.013**
Positive	2	3	
Negative	59	6	
*Mitotic figures* ≥ *3/10HPF*			***p* = 0.042**
Positive	1	2	
Negative	60	7	
*Periadrenal adipose tissue invasion*			*p* = 0.112
Positive	7	3	
Negative	54	6	
*Vascular invasion*			*p* = 0.625
Positive	9	2	
Negative	52	7	
*Capsular invasion*			***p* = 0.001**
Positive	11	7	
Negative	50	2	
*Profound nuclear pleomorphism*			*p* = 1.000
Positive	25	4	
Negative	36	5	
*Hyper-chromasia*			*p* = 0.625
Positive	9	2	
Negative	52	7	

Each PASS parameter was compared to *TOP2A* mRNA expression (high and low) using Fisher’s exact test

Significant *p*-values (< 0.05) are highlighted in bold

### Comparisons Between TOP2A mRNA levels, Metastases, and Survival

**Fig. 2:**
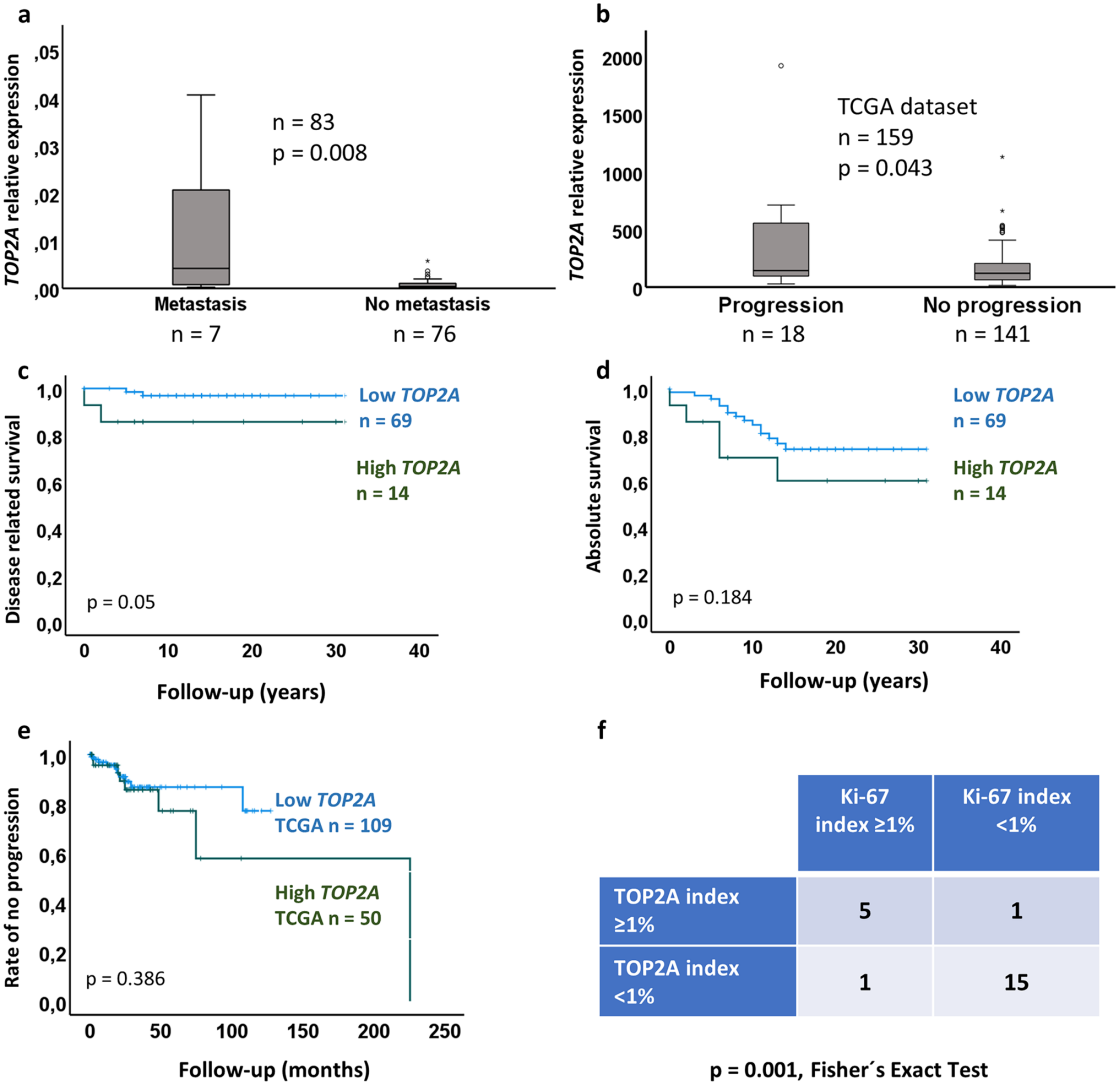
High *TOP2A* expression is associated with metastasis, shorter survival, and the Ki-67 proliferation index. **a** Relative expression of *TOP2A* in metastasizing PPGL (including local recurrences) compared to non-metastasizing PPGL. **b** Relative expression of *TOP2A* in tumors from the TCGA dataset, with progression of their disease (metastasis or relapse) compared to tumors with no progression. **c** Disease-related outcome in cases with *TOP2A* expression with values > mean (green) vs. < mean (blue) using log rank test. **d** Absolute survival in cases with *TOP2A* expression with values > mean (green) vs. < mean (blue) using log rank test. **e** Rate of no progression of tumors from the TCGA dataset, with *TOP2A* mRNA levels below vs above the mean value using log rank test. **f** Association between the immunohistochemical TOP2A and Ki-67 indices in 22 PPGL cases from our institution

Eight patients had presumed metastatic/recurrent disease (Supplementary Table 1). Two of these had germline *SDHB* mutations and one case exhibited an *EPAS1* mutation (not verified as either somatic or germline). Two cases exhibited somatic *KMT2D* mutations [33], of which one tumor also carried a somatic *NF1* mutation (Supplementary Table 1). PPGL cases with metastatic disease (or exhibiting local recurrence) displayed a significantly higher expression of *TOP2A* mRNA as compared to non-disseminated PPGL (*p* = 0.008, Fig. 2a, Table 1). Similarly, in the TCGA dataset, an association was found between high levels of *TOP2A* and progression (recurrence or metastasis as defined by TCGA in cBioportal) (Fig. 2b, Table 2).

In the survival analyses we observed an association between high *TOP2A* expression and shorter disease-related survival (time to death of disease) and a trend for shorter absolute outcome (time to death of any cause) (Fig. 2c, d; Table 1). In these analyses, cases were split into two groups, using values above (*n* = 14) and below (*n* = 69) the mean value of *TOP2A* mRNA expression. However, in the TCGA dataset, no significant associations were found for absolute outcome, disease-related outcome or measuring progression of disease using log rank test (Table 2, Fig. 2e).

To determine if *TOP2A* mRNA could be an independent predictor of metastatic disease, multivariate analysis was performed incorporating *TOP2A* together with the parameters tumor size, total PASS, age at diagnosis, and *TOP2A* mRNA relative expression. The analyses revealed that the *TOP2A* mRNA expression, and the parameters age at diagnosis and total PASS score were independent variables when predicting future metastasis (Supplementary Table 2).

### Immunohistochemical Detection and Localization of the TOP2A Protein and Comparison to Ki-67 Labeling Index

TOP2A immunostaining was performed for 37 PPGL cases. In addition, the clinical routine Ki-67 labeling index was successfully retrieved for a total of 22 cases in which TOP2A immunostaining was performed. A significant correlation between TOP2A and Ki-67 indices with a cut-off of ≥ 1% was noted (*p* = 0.001 Fisher’s exact test) (Fig. 2, Supplementary Table 1).

**Fig. 3:**
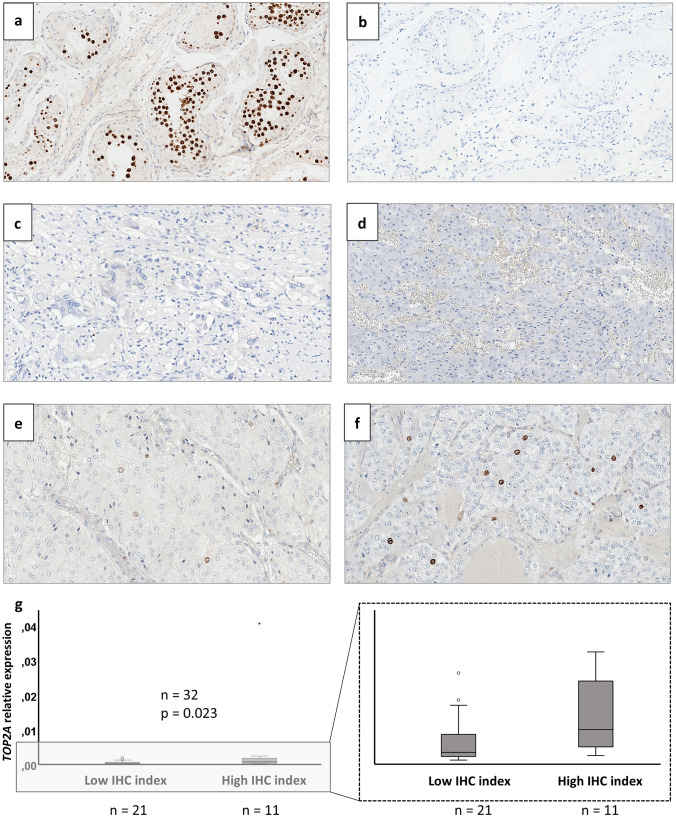
Detection and localization of TOP2A in pheochromocytoma and abdominal paraganglioma (PPGL) using immunohistochemistry. **a** Normal testicular tissue as positive control. Note the distinct nuclear signals. **b** Normal testicular tissue with omission of the primary anti-TOP2A antibody as a negative control. **c** Normal adrenal medulla serving as an additional negative control. **d** PPGL case no. 29 with a TOP2A index < 1%. **e** PPGL case no. 70 with a TOP2A index of 1%. **f** PPGL case no. 60 with a TOP2A index of 3%. **g** Relative expression of *TOP2A* in samples with a low immunohistochemical (IHC) index (< 1%) compared to expression in samples with a high IHC index (≥ 1%). Enlargement without outliers to the right

The immunostaining is illustrated in Fig. 3. Normal testicular tissue served as positive control, and was visualized as distinct nuclear signals (Fig. 3a). Normal testicular tissue with omission of the primary anti-TOP2A antibody and normal adrenal medulla served as negative controls, with no immunohistochemical expression of TOP2A (Fig. 3b, c). In the 37 PPGL cases analyzed for TOP2A expression by immunohistochemistry TOP2A index ranged from < 1% to 3.0%, averaging 1%, when measuring the proportion of tumor cells with nuclear staining (exemplified in Fig. 3d–f). Twenty-three cases exhibited a TOP2A index < 1%, two cases scored 1.0%, and 12 tumors exhibited scores > 1% (classified as “high” index cases). The TOP2A index for each immunostained case is available in Supplementary Table 1. Significantly higher *TOP2A* mRNA levels were found in the samples classified as “high” as compared to “low” immunohistochemical TOP2A index using Mann–Whitney *U*-test (*p* = 0.023, Fig. 3g).

### Copy Number Alterations and Methylation Density of the TOP2A Gene

To reveal a potential mechanism of TOP2A regulation, we investigated the TCGA dataset of 159 PPGL for genetic alterations such as mutations and copy numbers of the *TOP2A* gene. While no mutations were observed, a subset of cases had copy number alterations. Altogether 5 cases exhibited gain of *TOP2A*, 119 cases had no aberration, and 33 cases showed a loss of *TOP2A* (Table 2). Comparison of *TOP2A* mRNA levels in relation to copy number status showed no significant correlation (*p* > 0.1, Mann–Whitney *U*). Furthermore, data for methylation density at the eighteen available CpG sites of the *TOP2A* gene were investigated. Initially, potential correlations between levels of *TOP2A* and methylation in different CpG-sites were analyzed using Spearman’s test (Supplementary Fig. 1a). With regard to the mean methylation density at individual CpG sites, high methylation density was observed in the first and the second CpG site (Supplementary Fig. 1b), of which the first site showed a weak correlation to *TOP2A* mRNA levels. The other 16 CpG sites showed low mean levels of methylation, seven of which showed a weak inverse correlation to *TOP2A* mRNA levels.

## Discussion

The aim of this study was to investigate whether TOP2A could be used as a marker of poor clinical outcome in PPGL and distinguish metastatic tumors from non-metastatic ones. Indeed, TOP2A was found to be a potential marker of clinical importance since a significant association between elevated *TOP2A* mRNA expression and metastatic behavior was seen, which was also verified via analyses of non-institutional cases from the TCGA database. Additionally, associations of *TOP2A* mRNA expression to other clinical parameters, including death due to the tumor, were found. An association between *TOP2A* mRNA expression and TOP2A protein levels was also observed, suggesting that immunohistochemistry could be used to highlight cases with increased TOP2A levels — which is suitable for clinical purposes.

TOP2A has been suggested as a marker of poor prognosis and a potential therapeutic marker in several other cancer types, which make a similar mechanism in PPGL plausible. Similarly to the results presented in this study, Gong et al. [34] showed the potential role of TOP2A as a prognostic and therapeutic marker in papillary thyroid cancer as well as Chen et al. [35] did in renal cell carcinoma. Like Cluster 1 mutations in PPGL, hypoxia inducible factor (HIF) alterations due to *VHL* gene mutations are found in renal cell carcinoma, suggesting a potential similar molecular mechanism in PPGL. This is further discussed in another study where Jain et al. [18] suggested TOP2A as a therapeutic marker in metastatic and aggressive adrenocortical carcinoma. Indeed, high expression of TOP2A has been seen and associated with more aggressive behavior in various types of tumors [20, 21], indicating that high TOP2A expression may be a contributing factor to carcinogenesis.

In terms of association to histological parameters, there were no correlates between the total PASS score and high *TOP2A* expression in PPGL, even though an association between several, specific PASS parameters was found. The PASS algorithm was created for evaluating PCC and not aPGL/PPGL, and in this study high expression was found in mostly aPGL, which could have an impact on our results. However, the associations between *TOP2A* expression and the specific histological variables, mitotic figures, and capsular invasion indicate the potential of TOP2A as a marker of malignant outcome since these specific histological markers have been shown to be associated with malignant behavior by Kim et al. [32]. Potentially, these variables together with TOP2A expression could distinguish tumors with metastatic potential if reproduced in larger studies. Moreover, an association between *TOP2A* mRNA levels and immunohistochemical expression was noted, suggesting that the latter method could be of value in the clinical setting. As immunohistochemistry is currently the gold-standard technique for prognostic markers in most pathology laboratories, the findings obtained in this study could open up for a predictive marker used in the routine screening of PPGL. We also observed a correlation between TOP2A and Ki-67 indices in our series, which has been implicated by previous studies [27]. This finding is not surprising given the role of these two proteins as proliferation markers, but further emphasizes the association between increased cell growth and poorer outcome in PPGL.

We also wanted to investigate why TOP2A was elevated in tumors that later metastasized. Using the TCGA database, no *TOP2A* mutation in the coding sequence was observed, whereas copy number gain was noted in a few cases only without association to the *TOP2A* mRNA levels and methylation densities were only weakly correlated with *TOP2A* expression. This would imply that *TOP2A* mRNA is elevated due to other reasons, for example through regulation via transcription factors. Gupta et al. showed that there was a higher expression of TOP2A in malignant PPGL and could also see an association with loss of Retinoblastoma protein (pRB) expression [27]. Since *TOP2A* transcription is inhibited by pRB and p53, the observed association could make sense [36]. Similarly, an association between high TOP2A levels in cervical cancer and missense mutations in *TP53* and *RB1* was found by Yu et al. [37], implying the same type of mechanism. On the other hand, *TP53* mutations are very rare in PPGL which would contradict this idea. Overall, P53 and RB are well-known tumor suppressors in other cancer types, though their roles in PPGL are not completely known and need to be further investigated.

A significant association between high expression of *TOP2A* mRNA and Cluster 1 PPGL was also observed, indicating a possible connection between TOP2A and pseudo-hypoxia-related pathways. Since Cluster 1 tumors are considered most likely to metastasize, this further indicated the potential of TOP2A as a prognostic marker. The value of immunohistochemical markers pinpointing Cluster 1 disease cannot be emphasized enough for tumors with such a heterogenous genetic background, possibly allowing the pathologist to identify cases at risk of adverse clinical events in a timely fashion. Although CAIX and SDHB immunohistochemistry may triage subsets of cases, wider markers such as Alpha-inhibin (identifying Cluster 1 disease irrespectively of genetic background) have been recognized [38]. While TOP2A may be an additional marker in this context, it could also have implications from a therapeutic perspective, since Toh and Li showed that the topoisomerase II inhibitor Mitoxantrone inhibits the expression of HIF and hypothesized that it was due to an altered translation of HIF [16]. If the expression of HIF is dependent of the levels of TOP2A, PPGL tumors with Cluster 1 mutations could potentially be treated with topoisomerase inhibitors. HIF (and TOP2A) is elevated in several types of malignant tumors, which make this possible connection relevant in many other types of tumors as well. A recent study have shown an inhibitory effect on tumor growth with topoisomerase 1 inhibitors in, for instance, mouse PCC cell lines [39]. In that study, a decrease in HIF protein levels was also found, which is usually elevated in metastatic tumors, implying a positive effect of the drug. Additionally, Arivazhagan et al. [40] measured *TOP2A* mRNA levels in glioblastoma cells and observed that tumors with higher expression exhibited improved response from temozolomide treatment, a drug that is also sometimes used for metastatic Cluster 1 associated PPGL [41]. Today, there is no effective treatment for PPGL patients with metastatic disease, and further studies could be performed to evaluate topoisomerase inhibitors as an effective alternative. Topoisomerase 2 inhibitors, such as Mitoxantrone, could potentially be an effective alternative in disseminated PPGL, like it is used in breast cancer, lymphoma, and leukemia today [16].

Our study is not without limitations. First of all, the tumor cohort is limited, especially the number of truly metastatic cases, and hence any conclusions drawn between TOP2A and metastatic potential would need to be reproduced in a larger, independent material. This is especially true for patients with germline *SDHB* mutations, of which our cohort only could muster two such cases. Moreover, the mean TOP2A index has previously been reported as higher in metastatic cases [27]. As our metastatic cases scored somewhat lower than what has previously been reported, differences in methodology, patient demography, and overall scoring procedures may play a role here. Also, it is worth mentioning that among our PPGL cases defined as exhibiting malignant behavior, a single case could be vaguely suspected of misdiagnosis of a multifocal tumor rather than a locally recurrent event of the initial tumor, even though the patient’s recurrent tumor presented at the same site as the original tumor and was highly infiltrative with extension into nerve structures as well as adipose tissue.

We conclude that *TOP2A* mRNA expression is elevated in metastatic PPGL and associated to high-risk genetic aberrancies (*SDHB* and the *TERT* promoter), thus verifying the potential prognostic value of this marker. Moreover, *TOP2A* mRNA levels are associated with TOP2A and Ki-67 immunohistochemical indices, thereby supporting the utility of these markers in a clinical routine context. Additionally, the results from this study suggest that TOP2A may possibly constitute a therapeutic target using topoisomerase 2 inhibitors for a subset of metastatic cases, if reproduced in future functional and clinical studies.

## Supplementary Information

Below is the link to the electronic supplementary material.

Supplementary file1 (DOCX 232 KB)

Supplementary file2 (XLSX 32 KB)

## Data Availability

The datasets generated during the current study will be available upon reasonable request.
