# Supplementary material for: TOP2A Expression in Pheochromocytoma and Abdominal Paraganglioma: a Marker of Poor Clinical Outcome?
Source: Endocr Pathol. 2023 Jan 19;34(1):129–41. doi: 10.1007/s12022-022-09746-w (PMC10011289; doi:10.1007/s12022-022-09746-w)

**Supplementary Figure S1: Methylation density data and comparison to mRNA levels of *TOP2A* from TCGA. A.** Correlation analysis of *TOP2A* mRNA levels to methylation density at 18 CpG sites in the *TOP2A* gene using Spearman’s test. R-values are visualized in the figure, with statistically significant values marked (*). **B.** Mean methylation of each CpG site, CG site 1 showing the highest mean.


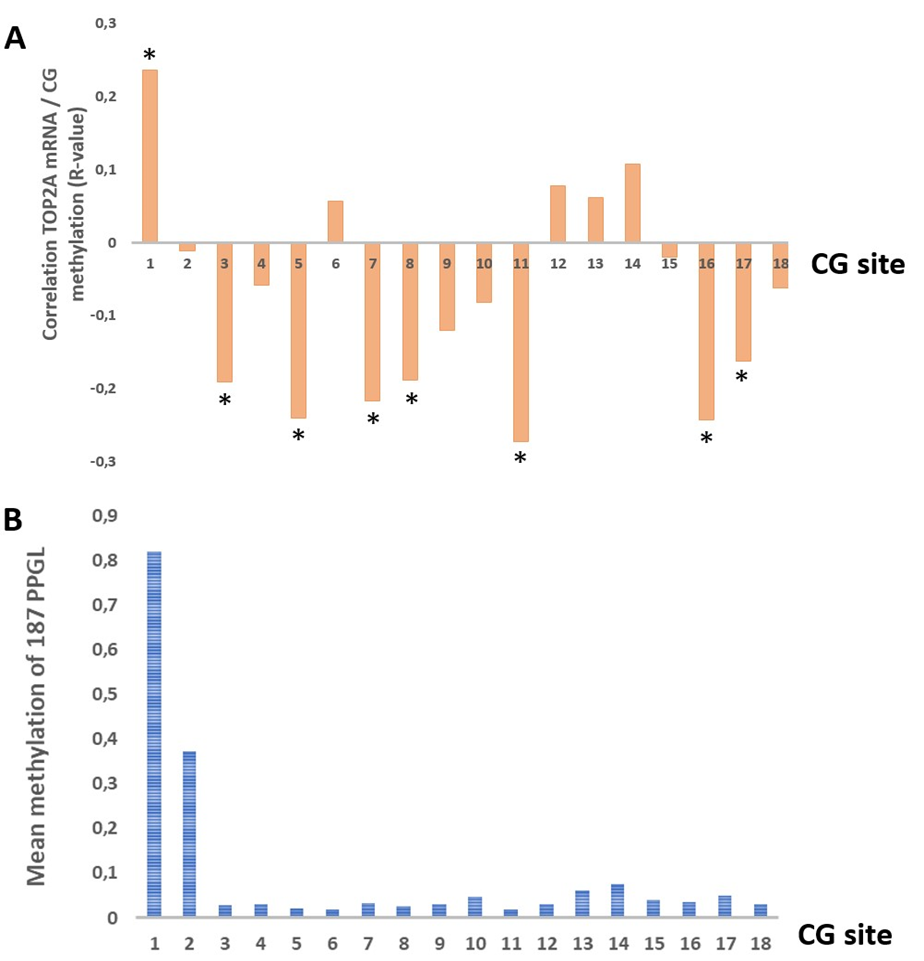

Supplement: Supplementary file 1 — Supplementary file1 (DOCX 232 KB) [file 12022_2022_9746_MOESM1_ESM.docx]
